# Atypical Presentation of Traumatic Pediatric Carotid Artery Dissection: A Case Report

**DOI:** 10.5811/cpcem.2022.4.56488

**Published:** 2022-08-06

**Authors:** Duncan McGuire, Nicholas Mielke, Amit Bahl

**Affiliations:** * Beaumont Hospital, Department of Emergency Medicine, Royal Oak, Michigan; † Oakland University William Beaumont School of Medicine, Department of Emergency Medicine, Royal Oak, Michigan

**Keywords:** carotid artery dissection, craniocervical artery dissection, pediatric trauma, ataxia, emergency medicine

## Abstract

**Introduction:**

Carotid artery dissection is a rare but serious condition manifesting with signs and symptoms that closely overlap with other more benign medical diagnoses. This vascular injury, however, can result in debilitating sequelae, including thromboembolic cerebrovascular accidents.

**Case Report:**

We describe the atypical presentation of a healthy eight-year-old male who presented to the emergency department (ED) with generalized abdominal pain and non-bloody, non-bilious emesis. These symptoms occurred nine days after he sustained blunt head trauma after a non-syncopal fall from standing while playing hockey. He was initially diagnosed with gastroesophageal reflux disease and constipation and was discharged home. The following day he developed an acute headache followed shortly by gait ataxia, prompting a return visit to the ED. Imaging of the head and neck revealed a left internal carotid artery dissection. The patient was started on intravenous unfractionated heparin and admitted to the hospital. He was later discharged symptom-free on therapeutic enoxaparin for eight weeks, followed by daily aspirin therapy.

**Conclusion:**

Pediatric trauma patients, especially those sustaining insult to the head and cervical spine, are at risk for craniocervical arterial injuries. This rare but dangerous pathology often manifests in a non-specific, delayed fashion making it a challenging diagnosis for physicians to make on the initial medical encounter.^1^,^2^ Maintaining a high clinical suspicion for carotid artery dissection is required to make this diagnosis and should guide a thorough history, physical examination, and appropriate imaging in order to improve patient morbidity and mortality. This case emphasizes key clinical features and risk factors of this disease that may help emergency clinicians promptly recognize and treat this entity.

## INTRODUCTION

Acute ischemic stroke (AIS) in the pediatric population is a relatively rare event, with an incidence estimated at around 2.5–8 per 100,000 children per year.^3^ Up to 20%^4^ of childhood AIS is due to craniocervical arterial dissection (CCAD), which can occur spontaneously, typically in an adolescent with known risk factors, but it most often presents after head and neck trauma.^3^ Craniocervical arterial dissection in children can lead to devastating neurological impairment and other long-term sequelae; so early recognition of CCAD in the pediatric population is crucial. In this case study, we present a young boy with a delayed presentation of post-traumatic CCAD.

## CASE REPORT

We present a case of a previously healthy eight-year-old male who fell while ice skating during a hockey scrimmage. The patient suffered a small scalp contusion, but there was no loss of consciousness, neurologic deficit, depressed Glasgow Coma Scale, subsequent seizure, or retrograde amnesia. He was not on anticoagulation. Nine days later, the patient presented to the emergency department (ED) with a chief complaint of diffuse abdominal pain and post-prandial, non-bloody, non-bilious emesis. He did not have any neurologic signs or symptoms at that time and was discharged from the ED with a diagnosis of gastroesophageal reflux disease and constipation. The following day he developed a headache followed shortly by an episode of acute ataxia resulting in difficulty with ambulation, prompting a return to the ED.

Upon repeat evaluation, the patient was well-appearing, in no acute distress, and well-hydrated. He was afebrile and hemodynamically stable (39.1 kilograms; temperature 36.5°C; heart rate 64 beats per minute; respiratory rate 24 breaths per minute; and blood oxygen saturation 100% on room air). On physical exam, he had an ataxic gait. The remainder of the exam was unremarkable. Given the acute neurological deficit, he underwent brain computed tomography (CT) without contrast, CT angiography (CTA) of the brain and neck, and magnetic resonance imaging (MRI) of the brain, which revealed a subacute left internal carotid artery dissection. With no known risk factors for spontaneous or traumatic vascular injury, this finding was presumed to be secondary to his prior non-syncopal fall. The patient was immediately started on an intravenous infusion of weight-based unfractionated heparin, which was later switched to subcutaneous enoxaparin. Neurosurgery was consulted and advised against acute surgical intervention. He was discharged symptom-free on therapeutic enoxaparin for eight weeks, followed by daily aspirin therapy. Subsequent repeat MRI several weeks later demonstrated spontaneous resolution of the intimal dissection.

## DISCUSSION

Craniocervical arterial dissection is an exceedingly uncommon event, with an annual incidence of 2.6–2.9 per 100,000 people. This pathology, however, accounts for up to 20% of strokes in young adults, a disproportionately large percentage.^4^ Vascular dissections can be spontaneous or traumatic in origin, with often innocuous mechanisms of injury. Craniocervical arterial dissection risk factors include connective tissue disorders that can predispose to vascular injury (eg, Ehlers-Danlos, Marfan’s syndrome, osteogenesis imperfecta, and fibromuscular dysplasia), blunt or penetrating head and neck trauma, male gender (53–57% of cases), migraine with aura, and hypercholesterolemia.^1^,^4^ Due to the spectrum of potential factors predisposing to dissection, it is critical to obtain a thorough medical history and perform a tailored diagnostic evaluation of patients found to have CCAD.

If clinical suspicion is high, magnetic resonance angiography (MRA), which possesses a sensitivity and specificity of 84% and 99%, respectively, is the recommended initial imaging modality.^5^ Alternative modalities include CTA (with sensitivity rates between 51–100% and specificity values 67–100%), Doppler ultrasound (sensitivity ranging between 71–96%), and conventional angiography.^6^,^7^ Conventional angiography is considered the gold standard for diagnosis of CCAD in pediatric and adult populations; however, potential complications including femoral hematoma, radiation exposure, and sedation requirement have contributed to the increasing popularity of MRI/MRA.^1^,^8^ In addition to being non-invasive and requiring no radiation exposure, MRI/MRA can simultaneously evaluate for stroke and dissection. Furthermore, this imaging modality can visualize intramural hematomas along arterial walls, which are estimated to occur in 76–91% of all vessel dissections.^1^

**CPC-EM Capsule**

What do we already know about this clinical entity?

*Early diagnosis of craniocervical arterial dissection (CCAD) in children is crucial given the potentially devastating neurological impairment and other long-term sequelae*.

What makes this presentation of disease reportable?

*This delayed and non-specific presentation emphasizes the importance that history, exam, and clinical suspicion play in the diagnosis of CCAD in children*.

What is the major learning point?

*Obtaining a history of trauma remains crucial, even in patients presenting with seemingly benign symptoms*.

How might this improve emergency medicine practice?

*This case emphasizes key clinical features and risk factors that may help emergency clinicians promptly recognize and treat CCAD*.

Patients with either spontaneous or traumatic CCAD often present with transient and nonspecific symptoms ranging from benign to severe in nature. Innocuous symptoms, which may include vomiting and neck pain, among others, can occur in the presence or absence of more concerning symptoms. One study reports that diffuse headache is more common than neck pain in children.^9^ Interestingly, the number of days from onset of headache or neck pain to neurological deficits is 15 hours and 14 days, respectively.^10^ Children with CCAD frequently present with neurologic findings; internal carotid artery dissection often results in the triad of ipsilateral neck and/or head pain, ipsilateral Horner syndrome, and delayed neurologic symptoms secondary to ischemia.^3^,^9^,^11^ Symptoms specific to ischemia of the anterior circulation include aphasia and hemiparesis, whereas posterior circulation ischemia most often results in ataxia, ipsilateral cranial nerve involvement, hemiplegia, and dysarthria. Cerebrovascular ischemic events can occur within minutes; however, they may not be present until up to one month after the initial insult.^12^ Patients who develop cerebral ischemia or infarction tend to have a good prognosis with nearly 75% regaining full functionality within 12 weeks.^4^

The case that we present highlights the tortuous course that may accompany diagnosing CCAD, emphasizing the importance that history and examination, and the clinician’s suspicion of this pathology play in this diagnosis. This suspicion should be increased in patients with nonspecific, delayed, post-traumatic symptoms and repeat ED visits. Although our patient did not develop any symptoms until nine days after his fall, eliciting a history of prior head and neck trauma during his initial ED presentation may have prompted the emergency physicians to consider a neurologic etiology for his seemingly benign symptoms of abdominal pain and emesis. It was not until the tenth day after his injury that he developed a headache and associated ataxia resulting in a thorough neurologic evaluation on repeat ED visit, which ultimately revealed the underlying diagnosis.

## CONCLUSION

Despite its rarity, craniocervical arterial dissection is the leading cause of cerebrovascular accidents in children. A wide variety of congenital and chronic diseases can predispose patients to CCAD; however, these conditions are not essential for this injury to occur. While symptoms related to CCAD can be immediate, many patients present in a delayed fashion. Clinicians must maintain a high index of suspicion when evaluating at-risk patient populations, recognizing that a benign mechanism of injury may cause this serious insult. This suspicion should be heightened when treating patients with repeat ED visits and should be in the differential when evaluating post-concussive patients, particularly those with a persistent or worsening headache or neurologic signs or symptoms.
